# PlantRG: A Comprehensive and User‐Friendly Database for Plant Resistance Gene Analogs (RGAs)

**DOI:** 10.1111/pbi.70691

**Published:** 2026-05-29

**Authors:** Jinghua He, Xiao Ma, Rui Cao, Zhuo Liu, Chenhao Zhang, Wei Chen, Lusheng Guo, Zipeng Meng, Rong Zhou, Xiaoming Song

**Affiliations:** ^1^ School of Life Sciences/School of Basic Medical Sciences/Library/Key Laboratory for Quality of Salt Alkali Resistant TCM of Hebei Administration of TCM North China University of Science and Technology Tangshan Hebei China; ^2^ State Key Laboratory of Southwestern Chinese Medicine Resources, Innovative Institute of Chinese Medicine and Pharmacy Chengdu University of Traditional Chinese Medicine Chengdu China; ^3^ College of Horticulture Nanjing Agricultural University Nanjing China; ^4^ Department of Food Science Aarhus University Aarhus Denmark; ^5^ National Center of Technology Innovation for Comprehensive Utilization of Saline‐Alkali Land Dongying China

## Abstract

Resistance genes are critical for plant defence against biotic stresses, and building a comprehensive, integrated data resource platform for these genes holds great significance for plant research and agriculture. Here, we developed PlantRG (http://plantrg.bio2db.com), a user‐friendly plant resistance gene database, which is built on 2 163 397 resistance genes identified from 1062 plant species. These genes were mined from all accessible plant genomic resources—systematically curated from 794 peer‐reviewed publications and 107 public databases—to ensure data breadth and reliability. All resistance genes in PlantRG were further functionally annotated using five major reference databases, enhancing their utility for targeted studies. Additionally, 207 353 SSR markers and 141 582 miRNAs associated with these resistance genes were detected, providing insights into their regulatory networks and genetic markers. Key bioinformatic results, including gene duplication patterns, protein–protein interaction predictions and CRISPR guide sequences, were also generated and stored in the database. PlantRG allows free browsing and downloading of all resistance gene sequences, annotations and bioinformatic data. It also offers practical tools such as Blast (for homology search), CasViewer (for CRISPR guide visualization), Circos (for genomic landscape analysis), HmmerSearch (for domain‐based identification) and Primer Design, to facilitate user‐friendly comparative genomic analysis. Notably, PlantRG is the comprehensive platform to complete large‐scale collection and bioinformatic analysis of plant resistance genes. It will support in‐depth studies on the structure, function and evolutionary patterns of resistance genes, thereby contributing to agricultural development—for example, breeding stress‐resistant crop varieties. In the future, PlantRG will be continuously updated to incorporate new data and features, maintaining its value for the global plant research community.

## 1 Introduction

Plant diseases represent a major factor in reducing crop yields, severely undermining global food security. According to studies (Savary et al. 2019), plant diseases cause yield losses of approximately 10.1%–28.1% for wheat, 24.6%–40.9% for rice, 19.5%–41.1% for maize, 8.1%–21.0% for potatoes and 11.0%–32.4% for soybeans. Such losses exert a significant impact on the global food supply. To tackle this challenge, researchers have conducted in‐depth investigations into the molecular mechanisms underlying plant diseases, aiming to develop effective plant disease prevention and control strategies. A key milestone in this field occurred in 1992: the resistance gene against *Helminthosporium carbonum* (a pathogen affecting maize) was identified via transposon tagging (Johal and Briggs 1992). This discovery marked the formal emergence of resistance genes in scientific research and laid the foundation for their widespread study. Subsequent studies have further confirmed that resistance genes play a crucial role in plant defence responses, providing novel insights for advancing research on disease prevention and control.

In nature, plants possess diverse structural and chemical defence mechanisms to fend off pathogens and pests (Ali et al. 2025; Ma et al. 2024; Tan et al. 2024). Among these, the two primary defence strategies that plants have evolved are as follows. One is the immune response triggered by pathogen‐associated molecular patterns (PAMP‐Triggered Immunity, PTI), and the second is the immune response triggered by effectors (Effector‐Triggered Immunity, ETI). In both plant defence responses (PTI and ETI), resistance genes play a crucial role (Boller and He 2009; Jones and Dangl 2006; Song et al. 2020). For the PTI defence response mediated by cell surface‐localized pattern recognition receptors (PRRs), receptor‐like kinase (RLKs) and receptor‐like protein (RLPs) serve as the major functional components. For the ETI defence response mediated by cytoplasmic resistance proteins, the primary functional molecules are NOD‐like receptors (NLRs). These NLRs can be further categorized into three subgroups: coiled‐coil (CC)‐nucleotide‐binding site (NBS)‐leucine‐rich repeat (LRR) (CNL), Toll/interleukin‐1 receptor (TIR)‐NBS‐LRR (TNL) and powdery mildew resistance 8 (RPW8)‐NBS‐LRR (RNL). These different categories of resistance genes are distinguished based on the encoded structural domains, which typically include CC (coiled coil), TM (transmembrane), NBS (Nucleotide‐binding adaptor shared by Apaf‐1, R‐proteins and CED‐4), TIR (Toll/interleukin‐1 receptor) domain, Kinase domain, LRR (leucine‐rich repeat) and RPW8 (Resistance to Powdery Mildew 8) domain. In addition to typical domains, resistance genes can also form fusion genes through domain rearrangement (Liu, Hou, and Chen 2024). These fusion genes often integrate multiple functional domains, such as Pkinase (Protein kinase domain), HMA (Heavy metal associated), WRKY (WRKY DNA‐binding domain), AP2 (APETALA2 domain), B_lectin (Bulb‐type Mannose‐binding Lectin) and PP2C (Protein phosphatase 2C), playing an important role in immune recognition, signal transduction, and disease resistance regulation. Through the coordinated functions of both canonical and fusion R genes, plants can construct a multi‐layered defence system, effectively resisting the invasion of pathogens and ensuring their growth, development and reproduction.

With the advancing development of sequencing technologies, an increasing number of plant species have been sequenced—providing abundant genomic data resources and expanding research avenues for studies on disease resistance genes (R genes). For example, the major resistance‐related *NBS‐LRR* family genes have been identified in most plants, such as *Triticum dicoccoides* (Boller and He 2009), *Oryza alta* *Swallen* (Shivute et al. 2024), *Euryale ferox* (Qian et al. 2022), *Actinidia chinensis* (Wang et al. 2020), *Saccharum spontaneum* (Jiang et al. 2023) and Rosaceae species (Guo et al. 2022). At the whole‐genome level, NBS‐LRR research has achieved comprehensive coverage across angiosperms (Yao et al. 2023). The *LRR‐RLK* family genes of *Arachis hypogaea* (Wang et al. 2024) and the *LRR‐RLP* family genes of *Musa acuminata* have been identified (Álvarez‐López et al. 2022). Furthermore, over 300 distinct R genes have been identified and cloned across the entire plant kingdom, with the majority belonging to the NBS‐LRR gene family (Gassmann et al. 1999).

To facilitate the exploration and utilization of plant resistance genes, several online data platforms have emerged, such as the Receptor‐like Kinase Database metaRLK (http://metaRLK.biocloud.top) (Liu, Fu, et al. 2024) and RLKdb (Yin et al. 2024) (https://biotec.njau.edu.cn/rlkdb) and the Plant Resistance Gene Database (PRGdb) (http://prgdb.org/prgdb4/) (Calle García et al. 2022). These platforms have greatly promoted the study of resistance mechanisms and have even provided important references for germplasm resource identification, evolutionary research and molecular breeding. However, facing the vast amount of resistance gene data, existing data platforms still cannot meet the needs for resistance gene identification and analysis, and there is a lack of a comprehensive and integrated data platform. Therefore, to enable researchers to obtain richer resistance gene data resources, we constructed this PlantRG platform. Users can obtain identification results and related analysis results of resistance genes in the database to promote the development of comparative genomics and functional genomics.

## 2 Results

### 2.1 Large‐Scale Analysis of Disease Resistance Genes Across Plant Genomes

**FIGURE 1:**
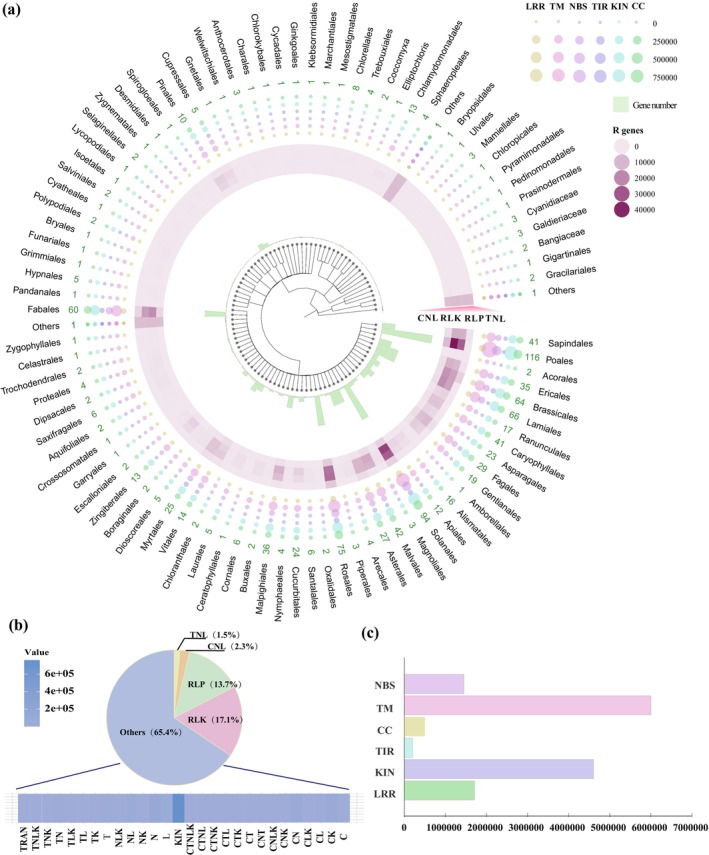
Distributions of disease resistance genes and their domains across plant species. (a) Circular chart showing the counts of total resistance genes, CNL, TNL, RLP, RLK genes and CC, TM, NBS, TIR, KIN, LRR domains across 94 plant orders (encompassing 1062 species). The green bar chart shows the total number of resistance genes. The purple heatmap illustrates the distribution of resistance gene families (*CNL*, *RLK*, *RLP* and *TNL*), with darker colour representing higher gene abundance. The circular heatmap depicts the distribution of CC, TM, NBS, LRR, KIN and TIR domains, with each domain represented by a ring ordered from the innermost to the outermost; larger ring size indicates higher domain counts. Numbers on the outer ring indicate the count of species. (b) Pie chart illustrating the proportional counts of *CNL*, *TNL*, *RLP*, *RLK* and other disease resistance gene. The blue square heatmap illustrates the abundance of non‐canonical gene families, where darker colours indicate higher gene counts. (c) Histogram displaying the number of NBS, CC, TM, TIR, KIN and LRR domains in all identified resistance genes.

We collected genomic information resources for 1062 plant species from 794 publications and 107 databases, and constructed a relatively comprehensive dataset (Figure 1a; Tables S1 and S2). Using these genomic resources, we identified disease resistance gene analogs (RGAs) via the DRAGO tool, yielding a total of 2 163 397 disease resistance genes (Table S1). These genes included 33 267 TNL (TIR‐NBS‐LRR) genes (1.5%), 50 157 CNL (CC‐NBS‐LRR) genes (2.3%), 296 017 RLP (receptor‐like protein) genes (13.7%), 269 806 RLK (receptor‐like kinase) genes (17.1%), and 1 414 150 analogs from other families (65.4%) (Figure 1b).

We further analysed the domain distribution of these resistance genes. Among the detected domains, transmembrane (TM) domains were the most abundant, totaling 6 003 886, whereas Toll/interleukin‐1 receptor (TIR) domains were the least common, with only 205 235 (Figure 1c; Table S3).

According to the identification results, the order Poales contained the highest number of disease resistance among all plant orders analysed (Figure 1). Within Poales, the sugarcane variety *Saccharum* spp. *R570* harboured the most identified RGAs (11801) across all examined species (Table S3). R570 is a polyploid sugarcane cultivar with a genome size of ~2C = 10 Gb and a karyotype of approximately 2*n* = 6*x* = 114. This complex polyploid genome structure is hypothesized to have increased gene copy numbers, thereby contributing to the elevated count of resistance genes in this variety (Healey et al. 2024).

Notably, the numbers of RGA analogs belonging to the RLK (receptor‐like kinase) and RLP (receptor‐like protein) families were higher than those of the CNL (CC‐NBS‐LRR) and TNL (TIR‐NBS‐LRR) families (Figure 1; Table S3). This phenomenon primarily stems from the multifunctionality of RLKs and RLPs: they not only mediate plant immunity (Song et al. 2020) and regulate growth and development (Liang and Zhou 2018) but also enable broad‐spectrum pathogen recognition (DeFalco et al. 2022; Ngou et al. 2024). These diverse roles likely drive plants to maintain a larger repertoire of RLK and RLP analogs to cope with complex environmental challenges, including pathogen infection and developmental regulation.

### 2.2 Contraction and Expansion Analysis of Disease Resistance Genes in Poales

Analysis of the contraction and expansion of *NLR* genes in Poales shows that this group as a whole exhibits a significant contraction trend. Among the species examined, there are obvious differences in the number of *NLK* genes, with *Saccharum sppR570* (2407), *Triticum aestivum* (2330) and *Dendrocalamus brandisii* (2194) ranking in the top three in terms of the number of *NLK* genes (Table S4). This lineage‐specific contraction trend, combined with differences in *NLK* genes abundance, indicates that Poales species have experienced different selective pressures during evolution, which may be closely related to their adaptation to diverse ecological and pathogenic environments (Ngou et al. 2022).

To further investigate the diversity and functional characteristics of *NLR* genes in the Poales, statistical analyses of canonical NLR domains (CC, NBS, LRR and TIR) and integrated domains (IDs) revealed that fusion domains were detected in 92 species. The abundances of NBS, LRR and CC domains showed a synergistic increasing trend, with the order of abundance being NBS > CC > LRR. As a representative monocot lineage, Poales exhibit a striking absence or severe depletion of TIR domains in NLR genes. In contrast, although the total number of integrated domains was much lower than that of NBS, LRR and CC domains, it was notably higher than that of TIR domains. This observation suggests that fusion genes may functionally compensate for the immune signalling pathways normally mediated by TIR domains (Andersen et al. 2020).

**FIGURE 2:**
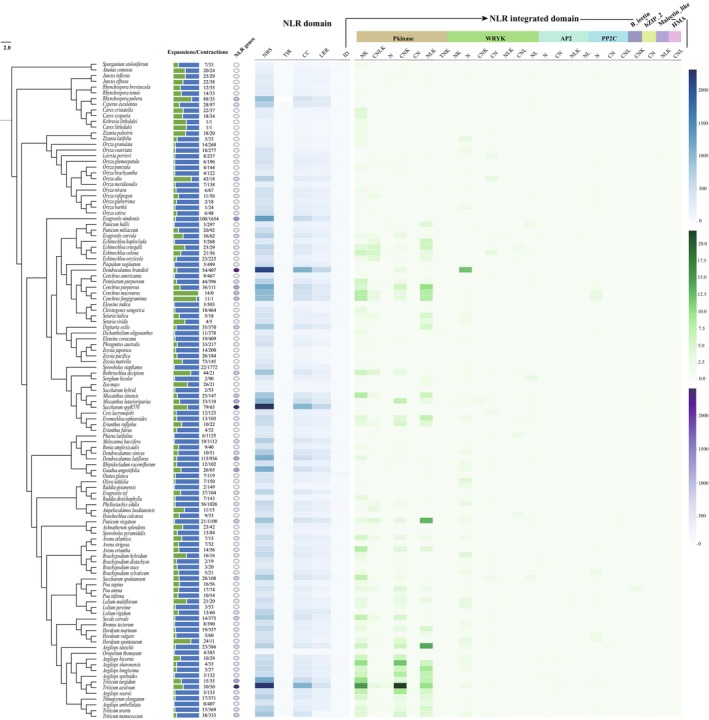
Statistics chart of *NLR* genes and fused disease resistance genes in poales plants. The proportional bar chart illustrates the relative abundance of expanded (green) and contracted (blue) gene families. A purple circular heatmap depicts the distribution of *NLR* genes across species. A blue rectangular heatmap shows the distribution of *NLR* genes harbouring canonical domains (CC, TIR, TM and NBS) and the non‐canonical domain Integrated Domains (ID). A green rectangular heatmap displays the distribution of *NLR* genes containing ID domains (Pkinase, WRKY, AP2, PP2C, HMA, B_lectin, bZIP_2 and Malectin_like) across CN, N, NL, CNL, NLK, NK, CNK, CNLK, TN, TNL, CNT, CTNL, TNK subfamilies. In all heatmaps, colour intensity correlates with gene count, with darker colours indicating a higher number of genes.

NLR‐IDs exhibit diverse distribution patterns in Poales (Figure 2; Table S4). Among them, fusion genes harbouring the Pkinase domain are significantly abundant, particularly in Triticum and its closely related species. WRKY‐type fusion genes are also highly prevalent in Poaceae, with the highest number detected in *Dendrocalamus brandisii*. In contrast, fusion events involving AP2, PP2C, HMA, bZIP_2 and B_lectin domains remain at relatively low levels across all examined species. *Triticum aestivum* possesses the most diverse repertoire of integrated domains, which is closely associated with its allohexaploid genome that has undergone multiple rounds of polyploidization and frequent genome rearrangements (Andersen et al. 2020; Bailey et al. 2018). The complex genomic background provides a higher probability of domain recombination and fusion events, thereby shaping its diverse complement of integrated domain combinations.

Notably, WRKY and PP2C domains show a strong integration preference for N‐type resistance genes lacking canonical CC and TIR domains. By contrast, AP2 domains display no obvious preference but are still abundant in N‐type genes. These observations suggest that such integrated domains may be directly involved in NBS‐mediated immune signalling activation. Furthermore, the abundance of these fusion domains is not significant in either CNL or TNL families, implying that functional constraints of core domains restrict the positions and modes of exogenous integration (Zeng et al. 2026).

### 2.3 Construction of the PlantRG Database

This study focuses on 1062 plant species. Genome, pest and disease, species images, taxonomy and reference information were obtained from authoritative databases such as NCBI, NGDC and CNSA and standardized to construct a foundational dataset. Disease resistance genes were identified by removing alternatively spliced sequences and using the DRAGO tool. Functional annotation was performed based on the Nr, Pfam, TrEMBL and Swiss‐Prot databases. Simultaneously, miRNA identification, gene duplication type analysis, SSR molecular marker development and CRISPR target design were completed to form a core disease resistance gene dataset.

**FIGURE 3:**
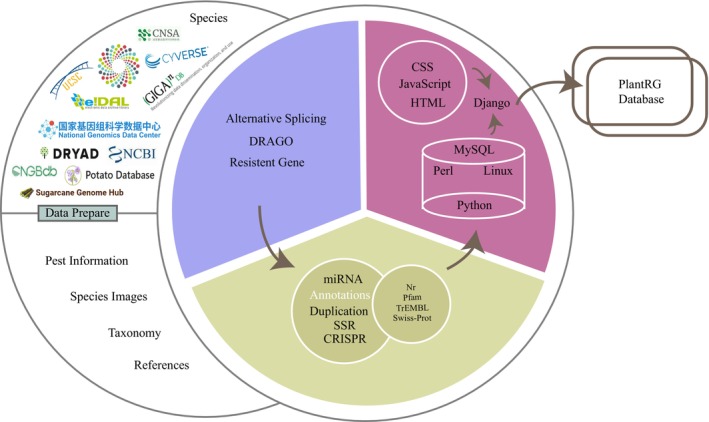
Workflow for PlantRG database construction. This figure illustrates the data collection, identification, annotation and integrated analysis pipeline employed to construct the core disease resistance gene dataset.

Supported by Linux, batch data processing was implemented using Python and Perl. A relational database was built using MySQL, and the frontend was developed with Django backend framework combined with HTML, CSS, and JavaScript. Ultimately, a plant disease resistance gene data resource sharing platform was established, integrating data query, visualization and batch download functions (Figure 3).

**FIGURE 4:**
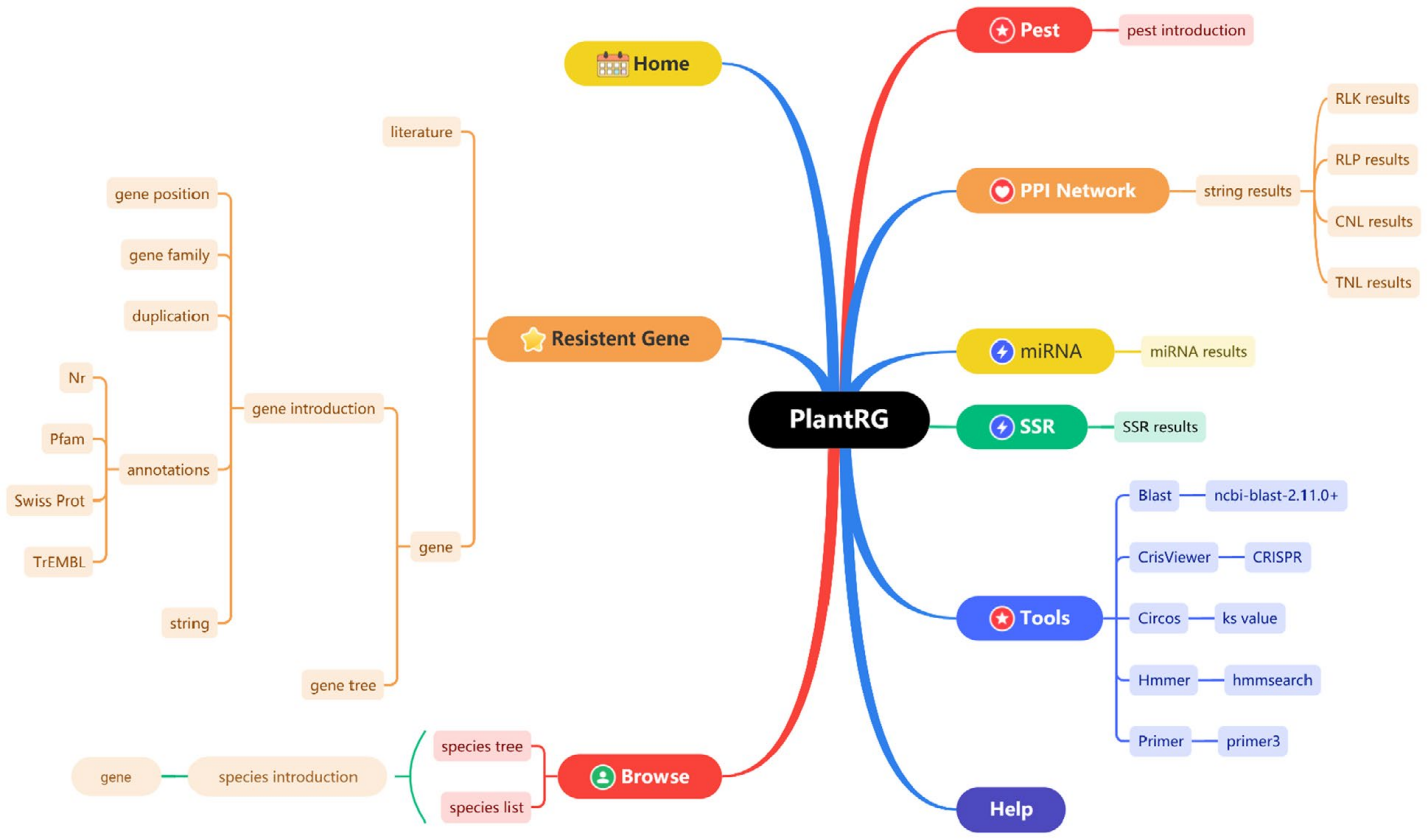
Architecture of the PlantRG database. The PlantRG database includes nine core modules: Browse, Download, Pest, PPI Network, SSR, miRNA, Resistant Gene, Tools and Help. Each module supports specific functions, from species and gene browsing, pest information, interaction and prediction results, to integrated analysis tools and usage guidance.

All data resources are stored in backend MySQL tables, which are readily accessible via the frontend web interface. We first provide an overview of the database's core interfaces, including Browse, Download, Pest, PPI Network, SSR, miRNA, Resistant Gene, Tools and Help (Figure 4). The Browse module supports species exploration via tree and list views, enabling users to quickly locate target species. The Resistant Gene module serves as the core of the database, integrating multi‐dimensional information including gene position, family classification, duplication type, functional annotations (Nr, Pfam, Swiss‐Prot and TrEMBL), protein interaction data, related literature and phylogenetic trees. The Pest module offers basic information about plant pathogens and pests, while the PPI Network module displays predicted interaction results for RLK, RLP, CNL and TNL gene families. The miRNA and SSR modules present corresponding prediction results, providing data support for regulatory mechanism research and molecular breeding. The Tools module integrates BLAST, CrisprViewer, Circos, HMMER and Primer3 to support downstream analyses. The Download module allows users to batch download data, and the Help module provides detailed usage instructions. The Help interface provides detailed usage tutorials and guidance, enabling users to quickly understand the database structure and efficiently use its core functions.

#### 2.3.1 Species Interface

**FIGURE 5:**
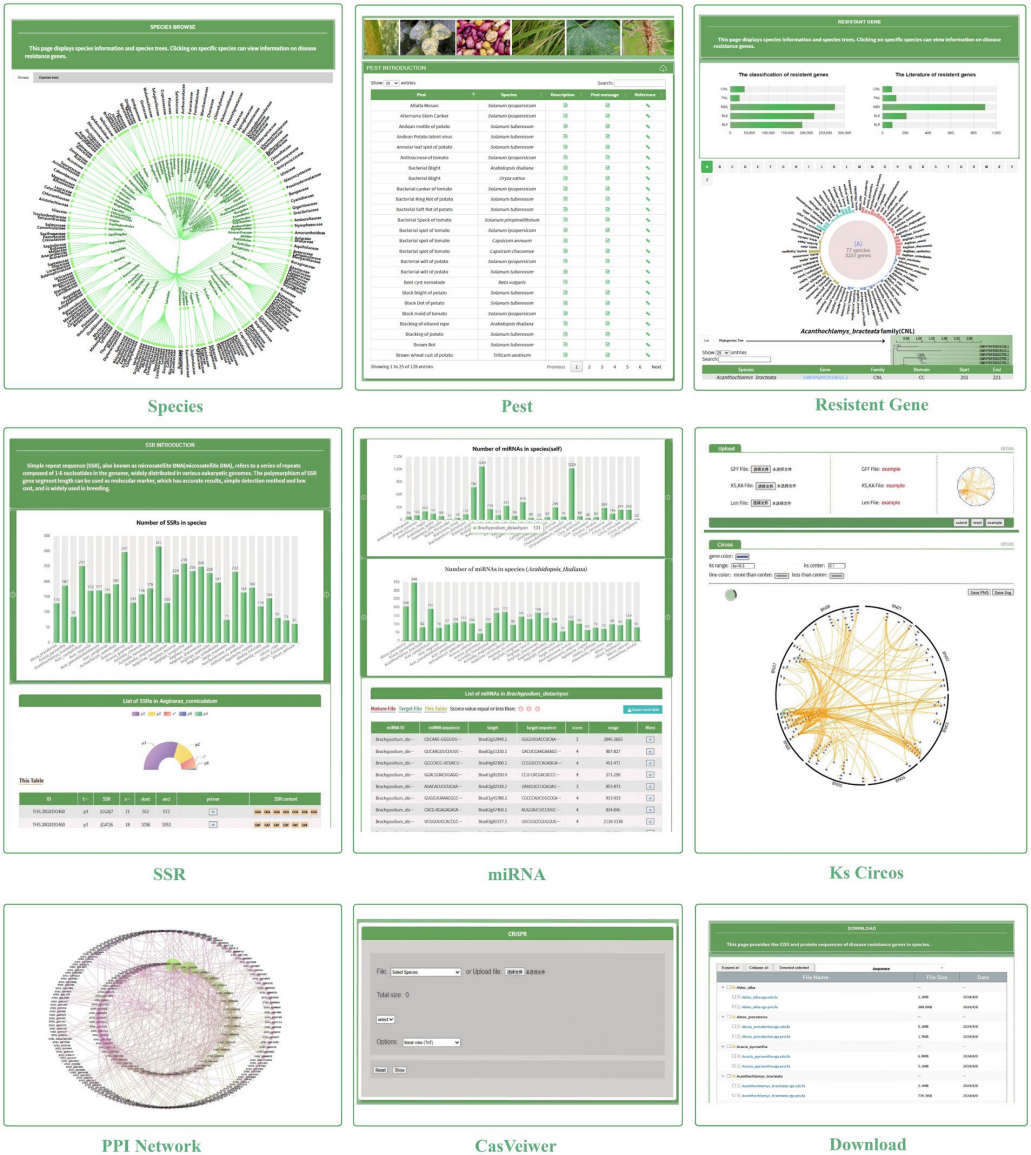
Main pages and their internal functions of the PlantRG database, including Browse, Pest, Resistant Gene, PPI Network, miRNA, SSR, Download and Tools pages.

The Species interface of PlantRG offers comprehensive information for included plant species, including their biological classification, species images, links to sequencing files, data download links, and details on species‐specific disease resistance genes (Figure 5; Tables S1 and S2).

The Tree section presents the biological classification of species clearly via an interactive tree diagram. By clicking on different classification nodes, users can conveniently locate and comprehend the classification information of specific species.

The Browse section showcases species images and names in a concise, intuitive format. Both species names and images are hyperlinked: clicking on either will display detailed classification and count information of disease resistance genes for the selected species.

#### 2.3.2 Pest Interface

The Pest interface of PlantRG displays comprehensive information for 109 disease types, including their basic symptoms, host plant species, detailed descriptions and associated reference messages.

Given the close association between disease resistance genes and plant diseases, we systematically curated information for these 109 diseases from 104 peer‐reviewed publications and integrated it into the database (Table S5). This resource enables users to quickly grasp the agricultural impacts of each disease—such as yield losses or host range—and further develop targeted prevention and control strategies to improve crop productivity.

#### 2.3.3 Overview of the Resistance Gene Interface

The Resistance Gene interface delivers detailed information on *CNL*, *TNL*, *RLP* and *RLK* resistance gene analogs (RGAs), covering key attributes such as RGA family classification, protein and CDS sequences, gene structure, functional annotation and duplication type.

Given the large number of resistance gene families in the database, this interface focuses specifically on the CNL, TNL, RLP and RLK families—groups with more extensive prior research. Data for other RGA families can be accessed via the Species interface for broader queries. To support intuitive data exploration: A histogram visualizes the count of RGAs across these four major families, enabling users to quickly grasp quantitative differences between categories. An interactive circular histogram is additionally provided to clarify the classification of each RGA type within different plant species. Clicking on a species name in this histogram displays all RGAs of the selected category (e.g., CNL) in that species. Complete information for each RGA (including sequences, annotations and duplication details) is accessible by clicking on its gene name. Furthermore, phylogenetic trees of these RGAs—constructed using the Muscle and FastTree tools—are visualized in this interface. This feature assists users in investigating the evolutionary relationships among resistance genes within a target species.

Gene duplication types are critical for investigating structural variations and functional differentiation of resistance genes. We identified duplication types for each resistance gene in the database, with five types detected: singleton, proximal, tandem, dispersed and WGD/segmental (whole‐genome duplication/segmental duplication).

**FIGURE 6:**
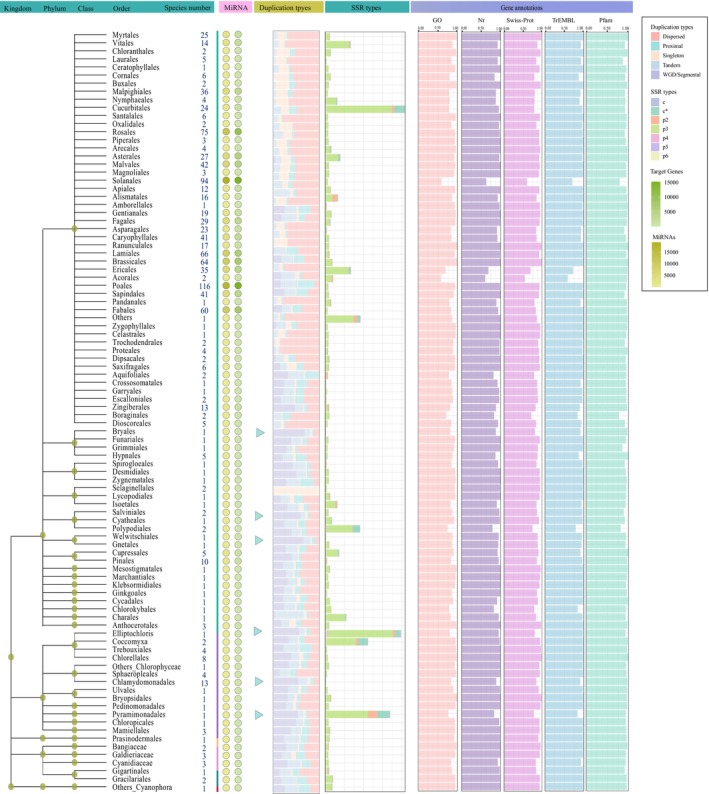
Visualization of bioinformatics analysis results for resistance genes, including the distribution of seven SSR (Simple Sequence Repeat) types in resistance gene analogs (RGAs), a heatmap of miRNAs and their corresponding target resistance genes, a pie chart showing the proportional distribution of five resistance gene duplication types (singleton, proximal, tandem, dispersed and WGD/segmental)—with triangles indicating orders where the WGD/segmental duplication proportion exceeds 50%—and a bar graph displaying the annotation percentages of all RGAs against five major databases (Nr: Non‐Redundant Protein Database, Swiss‐Prot, Pfam: Protein Family Database, TrEMBL and GO).

Among these, WGD/segmental duplication was dominant across many plant orders (Figure 6). For instance, the proportion of WGD/segmental duplication was highest in the order Crossosomatales (68%), followed by Acorales (65%). In Crossosomatales, *Euscaphis japonica* has undergone an ancient WGT (whole‐genome triplication) event and a recent WGD event (Xiao et al. 2023). In Acorales, *Acorus calamus* and *Acorus gramineus* share a common WGD event (Ma et al. 2023), while *A. calamus* has additionally undergone an ancient WGT event. These duplication events explain why the proportion of WGD‐derived resistance genes exceeds 50% in both orders.

Notably, *Eutrema japonicum* exhibits the highest proportion of WGD‐derived resistance genes (98.5%) (Table S6)—a result attributed to its shared WGD event with other members of the Brassicaceae family (Sun et al. 2021). Results of duplication type identification for individual resistance genes are accessible on their respective gene pages in the PlantRG database.

We completed functional annotations for all 2 163 397 resistance genes in the database based on five major protein databases. The annotation rates for these genes ranged from 63.34% to 100% (Table S7). All gene annotation information—including functional descriptions, conserved domain annotations and homologous protein matches derived from the five databases—can be accessed on the respective gene detail pages of the PlantRG database.

Additionally, to help users gain insights into the current research landscape of plant disease resistance, we have collected and curated all resistance gene‐related articles published in recent years from the NCBI database. These articles are systematically organized by resistance gene research categories to align with core research directions in the field.

For intuitive data interpretation, the publication count for each research category is visualized as a histogram, allowing users to quickly identify research hotspots. Meanwhile, detailed information of these publications—including article titles, authors, publication years, journals and links to NCBI abstracts/full texts—is presented in a clear list format, facilitating efficient retrieval of relevant literature.

#### 2.3.4 SSR Interface

Simple sequence repeat (SSR) marker technology is a primary tool for genetic mapping, cultivar fingerprinting, purity assessment and selection of trait‐specific molecular markers. It also plays a critical role in genetic research, cultivar identification, population genetic structure analysis, gene mapping and molecular‐assisted breeding—particularly in revealing genetic relationships among populations, where it provides key supportive data.

To leverage this technology for plant disease resistance research, we identified SSR markers for the disease resistance genes in PlantRG, with all resulting data stored in the database. A total of 207 353 SSRs were screened: among these, Chlamydomonas schloesseri had the highest SSR count (3023), while *Galdieria phlegrea* had the lowest (1) (Table S8).

Across all plant orders, trinucleotide (p3) SSR motifs accounted for a higher proportion than other SSR motif types (Figure 6). For user‐friendly access, SSR marker results for all species are visualized as bar charts (to show quantitative distributions) and provided in list format (for detailed data retrieval).

#### 2.3.5 miRNA Interface

MicroRNAs (miRNAs) are pivotal regulators of crop growth and defence, playing critical roles in modulating plant growth and development, as well as mediating plant disease resistance mechanisms. To support research on miRNA functions in plant disease resistance responses, we identified miRNAs associated with plant disease resistance from 1062 plant species using data from the miRBase and sRNAanno databases, yielding a total of 141 582 miRNAs (Table S1).

Among all plant orders, Solanales exhibited the highest counts of both miRNAs (19269) and their corresponding target resistance genes (15269) (Figure 6; Table S9). The PlantRG database also provides key predicted information for these miRNAs, including hairpin precursor sequences, mature miRNA sequences and their target resistance genes.

For intuitive data exploration, quantitative histograms (Figure 6) clearly display the number of miRNAs in each plant species. Visualizations of miRNA secondary structures (e.g., characteristic hairpin structures) are additionally provided to help users understand miRNA structural features.

All miRNA prediction results are presented in the miRNA interface of the PlantRG database and are available for direct download, facilitating further functional studies of miRNAs in plant disease resistance.

#### 2.3.6 Overview of the PPI Network Interface

Protein–protein interactions (PPIs) of disease resistance proteins (R‐proteins) provide valuable clues for understanding plant immune mechanisms. In this study, potential R‐protein PPIs across diverse plant species were computationally predicted using the STRING database with a stringent *E*‐value < 1e^−10^ cutoff. All predicted interactions were integrated into the PlantRG database and visualized as interactive networks (Figure 5). In the network diagrams, node size and colour reflect betweenness centrality, indicating the relative importance of individual R‐proteins. The interface allows users to browse interactive networks and customized subnetworks by R‐protein class and species.

Notably, these PPI data represent only computational predictions filtered by *E*‐value, which rely mainly on homology‐based inference from model organisms. Such predictions may have limited reliability for non‐model species without experimental validation and are intended for exploratory analysis only. We recommend combining these STRING‐based predictions with AlphaFold structural modelling to improve confidence in putative R‐protein interactions.

#### 2.3.7 Tool Interface

The PlantRG database integrates five specialized tools to support users in disease resistance gene (R gene) analysis: Circos, Blast, HmmerSearch, CasViewer and Primer Design.

Circos is a Ks (synonymous substitution rate) analysis result visualization tool built with D3.js, designed to help users investigate whether R genes are affected by polyploidization events or other types of duplication. To generate visualizations, users upload files in the specified example format: GFF files (gene structure annotation), CSV‐formatted Ks result files and chromosome sequence length files. The tool also allows users to adjust the Ks value range and customize gene colours—features that facilitate intuitive analysis and interpretation of Ks‐related data.

Blast & HmmerSearch tools support sequence alignment for coding sequences (CDS) or proteins, a core step in R gene homology analysis and functional annotation. Users perform alignments by uploading either a single sequence, a FASTA‐formatted sequence file or a designated database file (e.g., R gene sequence databases in PlantRG). This flexibility enables targeted searches for resistance gene analogs across different plant species.

CasViewer is specifically developed for visualizing CRISPR guide sequence files. It presents complex CRISPR guide sequence information (e.g., target sites, PAM sequences and guide RNA coordinates) in an intuitive graphical interface, helping users quickly identify key structural details of the CRISPR system. For pre‐stored CRISPR guide sequence detection results in PlantRG, users can directly view them by selecting the target species from a dropdown menu.

Primer Design is dedicated to designing primers for R gene amplification. Users upload the target R gene sequence or a FASTA‐formatted file containing the sequence; the tool then rapidly and accurately generates optimized primer pairs based on sequence characteristics (e.g., GC content and melting temperature). These primers are suitable for subsequent experiments such as PCR‐based gene cloning or expression analysis.

#### 2.3.8 Download and Help Interfaces

The PlantRG database includes dedicated Download and Help interfaces to optimize data accessibility and user experience, respectively.

The Download page enables users to retrieve and download disease resistance gene‐related sequences for all 1062 plant species in the database. All files are provided in FASTA format—a standard format compatible with most bioinformatics tools. To support large‐scale research, the interface facilitates convenient download of datasets covering all species at once, allowing users to quickly initiate comparative genomic analyses of resistance genes without repeated data collection.

The Help page is designed to lower the learning barrier for new users and ensure efficient platform navigation. It provides detailed, step‐by‐step usage instructions for every core interface of PlantRG (e.g., Species, Resistance Gene and PPI Network). Additionally, it includes a curated FAQ (Frequently Asked Questions) section addressing common operational issues (e.g., file upload requirements and query parameter settings). Together, these resources help users quickly grasp the database's functions and resolve potential challenges during use.

## 3 Discussion

With the increasing number of plant species with completed genome sequencing, the comprehensive identification and systematic analysis of resistance genes (R genes) in these species have become increasingly critical for advancing plant disease resistance research. In recent years, several plant R gene databases have been developed to address this need, including gabipD (http://www.gabipd.org/), PathoPlant (http://www.pathoplant.de/index.php) and PRGDB (http://prgdb.org/prgdb4/). While these platforms have provided valuable data resources for R gene studies, they are limited by relatively narrow species coverage and often lack the comprehensive data integration and analytical support required for in‐depth R gene research (e.g., incomplete downstream analysis results or insufficient annotation details).

Compared with these existing databases, PlantRG exhibits superior performance in four specific scenarios. First, PlantRG covers 1062 plant species, far more than the 233 species included in PRGdb, making it suitable for large‐scale cross‐species comparative genomic analyses. Second, the database integrates both typical and atypical disease resistance genes, supporting the mining of non‐canonical R‐genes. Third, PlantRG provides not only R gene identification results but also diverse downstream analytical outputs, including miRNA regulation, SSR markers, repetitive sequence identification, CRISPR guide sequences and gene duplication type annotations, enabling multi‐omics data integration and functional validation. Fourth, it focuses on non‐model plants, filling the gap that traditional databases are heavily biassed toward model species. In addition, PlantRG provides thorough functional annotations from five major protein databases (Nr, Swiss‐Prot, Pfam, TrEMBL and GO) and incorporates five user‐friendly online analytical tools (Circos, Blast, HmmerSearch, CasViewer and Primer Design) for in‐platform genomic analyses, which greatly streamlines research workflows.

Nevertheless, PlantRG currently lacks sufficient experimentally validated gene information. Therefore, it is more suitable as a valuable complementary resource for macroevolutionary analysis and multi‐omics integration studies, rather than a primary platform for direct breeding applications.

In summary, PlantRG stands out for its extensive species coverage, integrated analytical datasets, comprehensive R gene annotations and built‐in analytical tools. By filling critical gaps in existing R gene databases, it is well‐positioned to greatly advance plant R gene research—providing robust data support and practical analytical solutions for both plant pathology studies and crop resistance breeding. Ultimately, this resource will facilitate the deeper understanding of R gene function and evolution, and accelerate the development of disease‐resistant crop varieties to address agricultural challenges.

## 4 Materials and Methods

### 4.1 Collection and Retrieval of Resources

The genome sequence files, protein sequence files, coding sequence files and gff files were obtained through bioinformatics databases, including National Center for Biotechnology Information (NCBI, https://www.ncbi.nlm.nih.gov), GigaDB (http://gigadb.org/dataset/100331), (https://genomevolution.org/coge/), CNGB Sequence Archive (CNSA, https://db.cngb.org/cnsa/), PlantGIR (http://plantgir.cn) (Liu, Zhang, et al. 2024) and other genomics resource databases, such as TVIR (http://tvir2.bio2db.com), TAGR (http://tagr.bio2db.com), TBGR (http://www.tbgr.org.cn) and TGDF (http://tgdf.bio2db.com) (Liu, Huang, et al. 2024; Liu et al. 2022, 2025; Yu et al. 2025; Zhang et al. 2026). A total of 1062 species with the released sequences and images were collected, and the corresponding information of these species, including publication status, taxonomy, genome accession number, genome size and genome access databases.

### 4.2 Identification of Disease Resistance Gene Anologs

Disease resistance genes were identified using the DRAGO3 script written in the shell language (Calle García et al. 2022). Five major families of disease resistance genes have been identified, including *CNL*, *TNL*, *RLP*, *RLK* and other family genes. The identification of resistant gene duplication types was completed using the duplicate_gene_classifier program in the MCScanX software package (Wang et al. 2012). Fusion domain identification was conducted based on the following conserved domains: PF00069 (Pkinase), PF00403 (HMA), PF01453 (B_lectin), PP2C, PF00481 (AP2), PF00847 (BZIP_2), PF07716 (Melectin_like), PF12819 (WRKY) and PF03106. Only hits with an *E*‐value < 1e^−10^ were retained for further analysis.

### 4.3 Detection of SSR Markers

The SSRs in the resistance gene sequences of the selected species were identified using a program developed based on the Microsatellite Identification Tool (MISA), which also enables batch searching (von Stackelberg et al. 2006). Using the Primer3 program to design primers for identified SSRs (Rozen and Skaletsky 2000).

### 4.4 Functional Annotation of Resistance Genes and Detection of Duplication Types

Disease resistance gene annotations were conducted using five protein databases, including Swiss‐Prot and TrEMBL from the UniProt knowledgebase (https://www.uniprot.org) (Su et al. 2021), Pfam (v34.0) (http://pfam.xfam.org) (Mistry et al. 2021), Gene Ontology (GO, http://geneontology.org) and the non‐redundant protein sequence database (Nr, https://www.ncbi.nlm.nih.gov) (Li et al. 2025; Song et al. 2021).

### 4.5 miRNA Collection and Target Gene Identification

The mature, hairpin sequences and gff files of miRNAs were downloaded from sRNAanno and miRBase (Release 22.1) (Chen et al. 2021; Kozomara et al. 2019). The structure of each miRNA was created using the ViennaRNA package (v2.5.0) with slight modifications in batch (Lorenz et al. 2011). The target genes of each miRNA were predicted using the TargetFinder program (Kiełbasa et al. 2010), which applies the Smith–Waterman algorithm and a position‐dependent scoring matrix rather than simple sequence homology. Default parameters were used with a score threshold of ≤ 4.0, where penalties are doubled in the critical 5′ region (positions 2–13). While computational prediction may generate false‐positive outcomes, high‐confidence target genes can be effectively identified using more stringent score thresholds to reduce the false discovery rate. Nevertheless, key interactions still require rigorous experimental validation.

### 4.6 Guide Sequence Design for CRISPR

The CRISPR‐Cas9 targets were identified based on the CasFinder pipeline. First, repetitive genomic sequences were detected according to the previous report (Tarailo‐Graovac and Chen 2009). Then, the Bowtie program was used to create indexes for each genome (Ho et al. 2014). Finally, the two scripts, CasFinder.pl. and CasValue_v2.pl., were used to design guide sequences for CRISPR‐Cas9.

### 4.7 Construction of PlantRG Database

The PlantRG database takes the Django framework and MySQL database management system as the core to build its main website architecture according to the previous reports (Feng et al. 2024; Wang et al. 2025; Wu et al. 2024). It provides the Coding Sequences (CDS) and protein sequences of resistance genes, and conducts targeted bioinformatics analysis on these resistance genes. The MySQL database management system is utilized to store and manage the identified results and other related materials. By adopting the Django architecture and using HyperText Markup Language (HTML), Cascading Style Sheets (CSS), JavaScript and Python languages, interactive web pages can be built, and various query and search functions can be implemented. In addition, the Echarts data visualization tool is used to create various forms of pie charts or bar charts to achieve data visualization. D3 is used to draw protein interaction networks and circle diagrams.

## Author Contributions

X.S. conceived the project and was responsible for the initiation of the project; X.S., J.H., X.M. and R.C. supervised and managed the project and research. Data collection and bioinformatics analysis were performed by X.S., J.H., R.C., C.Z. and Z.M. Database construction was led by J.H., X.M., Z.L., W.C. and L.G. The manuscript was organized, written and revised by X.S., J.H., X.M., R.C., W.C. and R.Z. All the authors read and revised the manuscript.

## Funding

This work was supported by National Key Research and Development Program of China (2023YFF1002000), the National Natural Science Foundation of China (32172583), Tangshan Science and Technology Plan Project (24130219C), Tangshan Talent Funding Program (B202305014), the Basic Research Expenses for Provincial Universities (JJC2024001), Central Guidance and Support for Local Science and Technology Development Fund (246Z2509G), the S&T Program of Hebei (23372505D) and Hebei Natural Science Foundation (H2023209084).

## Conflicts of Interest

The authors declare no conflicts of interest.

## Supporting information


**Table S1:** Overall statistics of resistant gene anolgs and relevant bioinformatics analysis in 1062 species.
**Table S2:** Detailed classification and genome information of 1062 species.
**Table S2:** Statistics of resistant gene family and resistant gene domain in each species.
**Table S4:** Statistics of classic domains and fused domains of the NLK family in Poales plants.
**Table S5:** Detailed information of 109 disease in plants.
**Table S6:** Statistics of gene duplication type in each species.
**Table S7:** Gene functional annotation statistics of 1062 species using five databases.
**Table S8:** Statistics of SSR type in each species.
**Table S9:** Number of vegetable miRNAs in the sRNAanno and miRBase databases.

## Data Availability

All materials and data related to this study are available in our plantRG database (http://plantrg.bio2db.com).
